# Significant heterogeneity in *Wolbachia* copy number within and between populations of *Onchocerca volvulus*

**DOI:** 10.1186/s13071-017-2126-4

**Published:** 2017-04-18

**Authors:** Samuel Armoo, Stephen R. Doyle, Mike Y. Osei-Atweneboana, Warwick N. Grant

**Affiliations:** 1 Department of Animal, Plant and Soil Sciences, School of Life Sciences, La Trobe University, Bundoora, 3083 VIC Australia; 2 Environmental Biology and Health Division, Council for Scientific and Industrial Research, Water Research Institute, Accra, Ghana; 3 Present address: Parasite Genomics Group, Wellcome Trust Sanger Institute, Hinxton, Cambridge, CB10 1SA UK

**Keywords:** *Wolbachia*, *Onchocerca volvulus*, Onchocerciasis, Quantitative real-time PCR, Next-generation sequencing, Copy number, Heterogeneity

## Abstract

**Background:**

*Wolbachia* are intracellular bacteria found in arthropods and several filarial nematode species. The filarial *Wolbachia* have been proposed to be involved in the immunopathology associated with onchocerciasis. Higher *Wolbachia*-to-nematode ratios have been reported in the savannah-ecotype compared to the forest-ecotype, and have been interpreted as consistent with a correlation between *Wolbachia* density and disease severity. However, factors such as geographic stratification and ivermectin drug exposure can lead to significant genetic heterogeneity in the nematode host populations, so we investigated whether *Wolbachia* copy number variation is also associated with these underlying factors.

**Methods:**

Genomic DNA was prepared from single adult nematodes representing forest and savannah ecotypes sampled from Togo, Ghana, Côte d’Ivoire and Mali. A qPCR assay was developed to measure the number of *Wolbachia* genome(s) per nematode genome. Next-generation sequencing (NGS) was also used to measure relative *Wolbachia* copy number, and independently verify the qPCR assay.

**Results:**

Significant variation was observed within the forest (range: 0.02 to 452.99; median: 10.58) and savannah (range: 0.01 to 1106.25; median: 9.10) ecotypes, however, no significant difference between ecotypes (*P =* 0.645) was observed; rather, strongly significant *Wolbachia* variation was observed within and between the nine study communities analysed (*P* = 0.021), independent of ecotype. Analysis of ivermectin-treated and untreated nematodes by qPCR showed no correlation (*P =* 0.869); however, an additional analysis of a subset of the nematodes by qPCR and NGS revealed a correlation between response to ivermectin treatment and *Wolbachia* copy number (*P* = 0.020).

**Conclusions:**

This study demonstrates that extensive within and between population variation exists in the *Wolbachia* content of individual adult *O. volvulus*. The origin and functional significance of such variation (up to ~ 100,000-fold between worms; ~10 to 100-fold between communities) in the context of the proposed mutualistic relationship between the worms and the bacteria, and between the presence of *Wolbachia* and clinical outcome of infection, remains unclear. These data do not support a correlation between *Wolbachia* copy number and forest or savannah ecotype, and may have implications for the development of anti-*Wolbachia* drugs as a macrofilaricidal treatment of onchocerciasis. The biological significance of a correlation between variation in *Wolbachia* copy number and ivermectin response remains unexplained.

**Electronic supplementary material:**

The online version of this article (doi:10.1186/s13071-017-2126-4) contains supplementary material, which is available to authorized users.

## Background

Onchocerciasis, also known as river blindness, is a human parasitic disease caused by the filarial nematode *Onchocerca volvulus.* The disease is estimated to affect approximately 37 million people worldwide [1], and is associated with a range of dermal and ocular pathologies [2]. As is also the case for many arthropods, *O. volvulus* harbour endosymbiotic *Wolbachia* bacteria [3, 4] and, in *O. volvulus*, it is proposed that the bacteria, rather than the nematode itself, drive the immunopathology associated with the disease [5–7]. It is also proposed that *Wolbachia* are essential for worm survival [8–11] and are thought to play an important role in several biosynthetic pathways during reproduction and growth of their filarial worm hosts [12]. It was the observation of a correlation between the gradual loss of *Wolbachia* and reductions in the fecundity and viability of adult worms following treatment with tetracyclines that suggested (a) that targeting *Wolbachia* may offer an avenue to development of a macrofilaricide, and (b) that unlike *Wolbachia* in arthropods (which are pathogens), the *Wolbachia* of filarial worms may be essential and beneficial symbionts [10, 13, 14].

A longstanding and broadly accepted hypothesis is that there are at two strains or “ecotypes” of *O. volvulus*, and that these two ecotypes correlate with two distinct patterns of pathology [15–17]. The forest ecotype causes a mild form of the disease that presents mainly as dermal rather than ocular pathology [16], whereas the savannah ecotype is associated with the severe ocular form of the disease [17]. Although there is currently no mechanistic explanation for the different outcomes of infection, a previous study identified a correlation between *Wolbachia* copy number and ecotype, whereby the density of *Wolbachia* per nematode in a sample of savannah ecotype parasites was significantly higher than in similar sample drawn from the forest ecotype [18]. This correlation was interpreted as support for the hypothesis that higher *Wolbachia* densities were associated with the more severe disease pathology of the savannah ecotype [18]. However, the DNA samples that were analysed by Higazi et al. [18] were prepared from whole, intact nodules that contained an unknown mix of adult worms of both sexes and microfilariae (in utero in females and in the tissue of the nodules), and it is not clear what *Wolbachia* density means in this context. Furthermore, although the authors of that study noted the occurrence of variation between the communities studied, the data from several communities from each ecotype were aggregated and any variation that may have occurred within and between communities (or between individual patients/nodules) was not reported. Finally, deviations from the forest-savannah, mild-severe disease dichotomy clearly exist: isolated forest regions with high incidence of blindness correlating with microfilaria intensity have been reported [19]. On a genetic level, alleles of the O-150 DNA repeat have been defined as a means by which *O. volvulus* forest and savannah populations can be differentiated [20, 21], although novel O-150 alleles have been identified that do not fit the “classical” forest-savannah categorization [22, 23], and the increasing availability of information on genetic variation from genomic data is uncovering further population stratification that is not consistent with this simple dichotomy (Doyle & Grant, in preparation). It seems possible, therefore, that a classification of parasite pathogenicity based on forest and savannah ecotypes and the correlation between ecotype and *Wolbachia* density may be oversimplified, and that it may be informative to re-examine *Wolbachia* copy number with the focus on analysis of variation at the community and individual worm levels, as well as by ecotype.

In this study, we have focussed attention on the intra- and inter-community variation of *Wolbachia* copy number relative to their host using a quantitative PCR (qPCR) assay and next-generation sequencing (NGS). In addition, we investigated the density of *Wolbachia* in host worms from the two ecotypes. We have also examined the effect of ivermectin (IVM) exposure and drug-response on *Wolbachia* copy number. This examination was prompted by two pieces of evidence: first, IVM treatment could influence the relative copy number of *Wolbachia* genomes in *Dirofilaria immitis* [24], and second, a preliminary analysis of *O. volvulus* NGS data from pools of ivermectin (IVM) treated and untreated worms discovered a higher proportion of *Wolbachia-*associated reads from IVM-treated worms compared to untreated worms (Additional file 1: Table S1).

## Methods

### Sample preparation

**Fig. 1:**
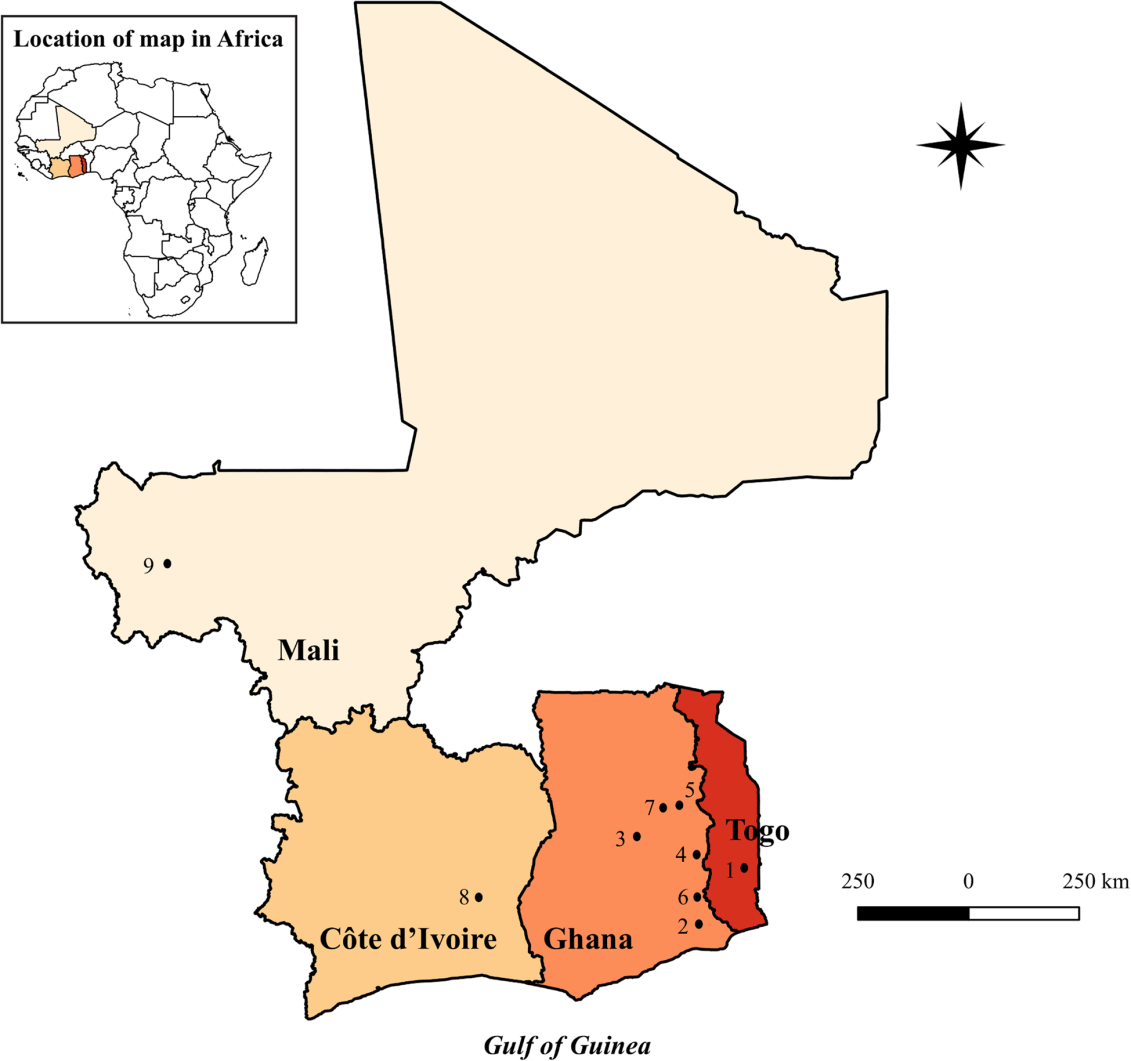
Map of West Africa showing study countries and communities. Communities have been numbered by alphabetical order from the East to West. Togo: 1, Tadome. Ghana: 2, Aflakpe; 3, Asubende; 4, Asukawkaw; 5, Jagbengbendo; 6, Todzi; 7, Wiae. Côte d’Ivoire: 8, Grobaledou. Mali: 9, Koudian

A total of 234 male and female adult *O. volvulus* samples (Table 1), which were obtained by the surgical excision of nodules from infected patients living in 9 communities in the forest and savannah transmission zones of 4 countries: Togo, Ghana, Côte d’Ivoire and Mali (Fig. 1), were used in this study. The samples were obtained from a broad geographical range of communities within the Onchocerciasis Control Program region of West Africa and differed in their exposure to IVM at the time of sample collection. DNA was extracted from entire males and from the anterior or posterior 4 cm of females (that do not contain reproductive organs), to avoid variation in estimation of the *Wolbachia* to nuclear ratios that may be due to variation in the reproductive activity of female worms. An additional set of DNA samples (38 in all) were obtained from the non-reproductive tissues of female adult worms that had been subjected to embryogram analysis to determine their reproductive status post-ivermectin treatment and were classified as good or sub-optimal responders depending on the presence of live embryos and/or microfilariae in utero [25–27]. *Onchocerca volvulus* adults were extracted from nodules by collagenase digestion followed by gentle teasing of the digested nodules to isolate individual nematodes [28]. Genomic DNA was prepared from single nematodes using the DNeasy® Tissue kit (Qiagen, Hilden, Germany) following manufacturer’s instructions.

**Table 1:** Breakdown of samples by country, community, ecotype and ivermectin treatment history. Samples were collected from 9 communities in 4 countries in West Africa

Country	Study community	Number of samples	Ecotype	IVM treatment history
Togo	Tadome	34	Forest	Untreated
Ghana	Aflakpe	19	Forest	Untreated
	Asubende	30	Savannah	Treated
	Asukawkaw	23	Forest	Treated
	Jagbengbendo	48	Savannah	Treated
	Todzi	21	Forest	Treated
	Wiae	29	Savannah	Treated
Côte d’Ivoire	Grobaledou	17	Forest	Untreated
Mali	Koudian	13	Savannah	Treated

### Quantitative real-time PCR assay development and validation

A qPCR assay was designed to measure the relative number of *Wolbachia* genome(s) per nematode genome, like those employed by others [18, 24, 29]. To develop this assay, single copy genes per genome were used: the *Wolbachia* surface protein (*wsp*) gene (GenBank Accession: HG810405.1) and the *O. volvulus* glutathione reductase (*gr*) gene (GenBank Accession: Y11830.1) were selected. Primers for *wsp* (Forward: 5′-AAC CGG GAC AAA AAG AAG AG-3′; reverse: 5′-CAG CAA CCT ACC AAA GAT GGA-3′; 110-bp product) and *gr* (forward: 5′-GTG CGA CGA AGA AGG ATT TC-3′; reverse: 5′-GCT TAT GCT GTT TCG GGT TT-3′; 103 bp product) were designed using CLC Genomics Workbench 6.5 (CLCbio, Aarhus, Denmark). Both primer sets were designed within well-conserved regions of the genes. Also, to ensure uniqueness of the primer regions to our target genes, we screened the primer regions against the complete *Wolbachia* nuclear (GenBank Accession: HG810405.1) and mitochondrial (GenBank Accession: AF015193.1) (NCBI BioProject Accession: PRJEB513; GenBank Assembly Accession: GCA_000499405.2) reference genome assemblies of *O. volvulus* by using the BLAST tool in CLC Genomics Workbench 6.5 (CLCbio, Aarhus, Denmark).

Each qPCR reaction mixture (total volume: 10 μl) included 0.2 μM of each primer, 2 μl of DNA and 5 μl of SsoAdvanced™ Universal SYBR® Green Supermix (Bio-Rad Laboratories Inc., California, USA). All qPCR runs were performed in duplicate on a CFX 96 Real-Time PCR Detection System (Bio-Rad Laboratories Inc., California, USA). The reaction conditions were optimised by gradient PCR, from which the optimal conditions were found to be as follows: an initial incubation at 95 °C for 2 min, followed by 40 cycles of 95 °C for 5 s, 53.8 °C for 15 s and 72 °C for 15 s. Melt curves were generated at the end of each run to ensure specificity of the amplification (Additional file 2: Figure S1c, g), which were performed as follows: 95 °C for 30 s, annealing at 65 °C and increments to 95.5 °C, holding for 5 s at each step of increment. Amplification efficiencies (E) for each primer set (Additional file 2: Figure S1a, b) were determined by generating a 10-fold dilution series across 8 orders of magnitude (starting at 1 ng), using cloned PCR product in pGEM®-T Easy vector (Promega Corporation, California, USA). Standard curves were also generated for each primer set, using *O. volvulus* genomic DNA (Additional file 2: Figure S1e, f) to determine the maximum quantification cycles (Cq) that could be used for data analysis, from which a cut-off Cq value of 30 was determined to be the Cq threshold before significant technical inconsistencies were observed. The number of *Wolbachia* genome(s) per nematode genome was determined using the following equation per sample:

Relative copy number (*wsp*/*gr*) = 2^([*μ*Cq. *gr* * E. *gr*] − [*μ*Cq. *wsp* * E. *wsp*])^

where μCq is the mean amplification cycle for each target, and E is the reaction efficiency for each target (determined by measuring the slope across the linear range from the standard curves described above).

### Relative copy number estimation by next-generation sequencing

The relative copy number of *Wolbachia* genomes was also estimated for an additional 38 adult worms for which the reproductive response to ivermectin was known. The full methods and data for sequencing of these worms will be published elsewhere. Briefly, genomic DNA for individual worms was sheared to an average fragment length of 400 bp, and barcoded Illumina sequencing libraries for each individual worm were prepared using the NEBNext® Ultra™ DNA Library Prep kit for Illumina (E7370L, New England Biolabs, Inc. Ipswich, USA). The barcoded single worm libraries for up to 16 worms were pooled per Ilumina HiSeq lane and paired-end sequenced (Illumina Inc., San Diego, USA). After standard trimming to remove adaptor and barcode sequences, and filtering for quality using Trimmomatic [30], BWA-MEM software [31] was used to map reads to the *Wolbachia* nuclear and mitochondrial reference genomes of *O. volvulus*. Relative *Wolbachia* copy number was calculated as the ratio of the number of bases mapped to the *Wolbachia* genome to the number of bases mapped to two major scaffolds of the nuclear genome.

### Statistical analyses

Data analyses, including Wilcoxon rank sum test (*W*) and Kruskal-Wallis rank sum test (*K-W*), were performed in the programming language, R version 3.2.2 [32]. *P-*values < 0.05 were considered significant. Microsoft Excel (2011) was used for all other data analyses.

## Results and discussion

**Fig. 2:**
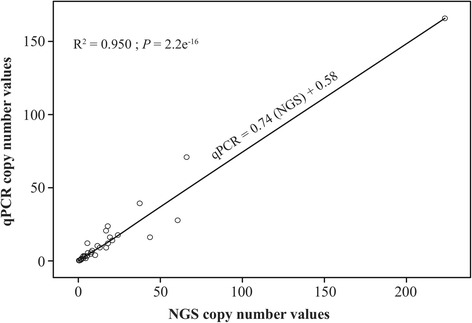
Correlation between qPCR and NGS copy number estimates. Observed a significant positive correlation between these estimates (*R* ^2^ = 0.950, *F* = 602.5, *df* = 31, *P* < 2e^-16^)

A total of 92 females and 142 male worms were analysed by qPCR to determine the *Wolbachia* genome to *O. volvulus* nuclear genome copy number ratio, which was used as a proxy for the relative number of *Wolbachia* per nematode. An initial examination of sex bias demonstrated that there was not a significant difference in *Wolbachia*: nuclear copy number ratio between male and female worms (Additional file 3; Figure S2; *W* = 7,079, *df* = 1, *P* = 0.668), which is consistent with previous studies in other filarial nematodes [24, 29]. As an independent means to verify the data obtained by qPCR, we used NGS to derive the relative copy number of the two genomes by calculating the mean read depth of the two genomes. A high correlation was found between the copy number ratios obtained from the qPCR and NGS datasets (Fig. 2; *R* ^2^ = 0.950, *F* = 602.5, *df* = 31, *P* < 2e^-16^), demonstrating that our qPCR assay was sensitive and specific.

**Fig. 3:**
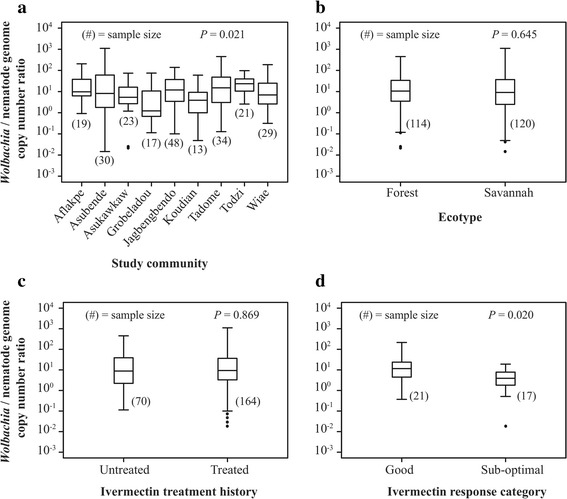
Comparisons of *Wolbachia*: nematode genome copy number ratios among different categories of worms. Box and whisker plot shows the median (line within box), the 25^th^ and 75^th^ percentile (lower and upper limits of the box respectively). The whiskers indicate the 10th and 90th percentiles, with outliers represented by *dark dots*. **a** Comparison among study communities (*P*-value of 0.021 from Kruskal-Wallis rank sum test). **b** Comparison between forest and savannah ecotypes (*P*-value of 0.645 from Wilcoxon rank sum test). **c** Comparison between IVM-treated and untreated worms (*P*-value of 0.869 from Wilcoxon rank sum test). **d** Comparison between good and sub-optimal responders to ivermectin (*P*-value of 0.020 from Wilcoxon rank sum test)

Analysis of *Wolbachia*: nuclear copy number ratios based on communities of origin of individual host worms, demonstrated that there was remarkable within community variation (Fig. 3a); the upper and lower 90^th^ percentile boundaries for all communities ranged over ~ 10–100-fold, with up to ~ 100,000-fold differences between the upper and lower extremes within each community. An example of significance within community variation is seen in worms from Asubende, a savannah community in Ghana, in which the highest (1,106.2) and lowest (0.01) individual worm *Wolbachia*: nuclear copy number ratios were recorded (median: 8.15). Such extreme variation has not been previously described, however, a wide range of *Wolbachia* copy number values in *O. volvulus* and other filarial nematodes have been reported previously [18, 24, 29].

**Fig. 4:**
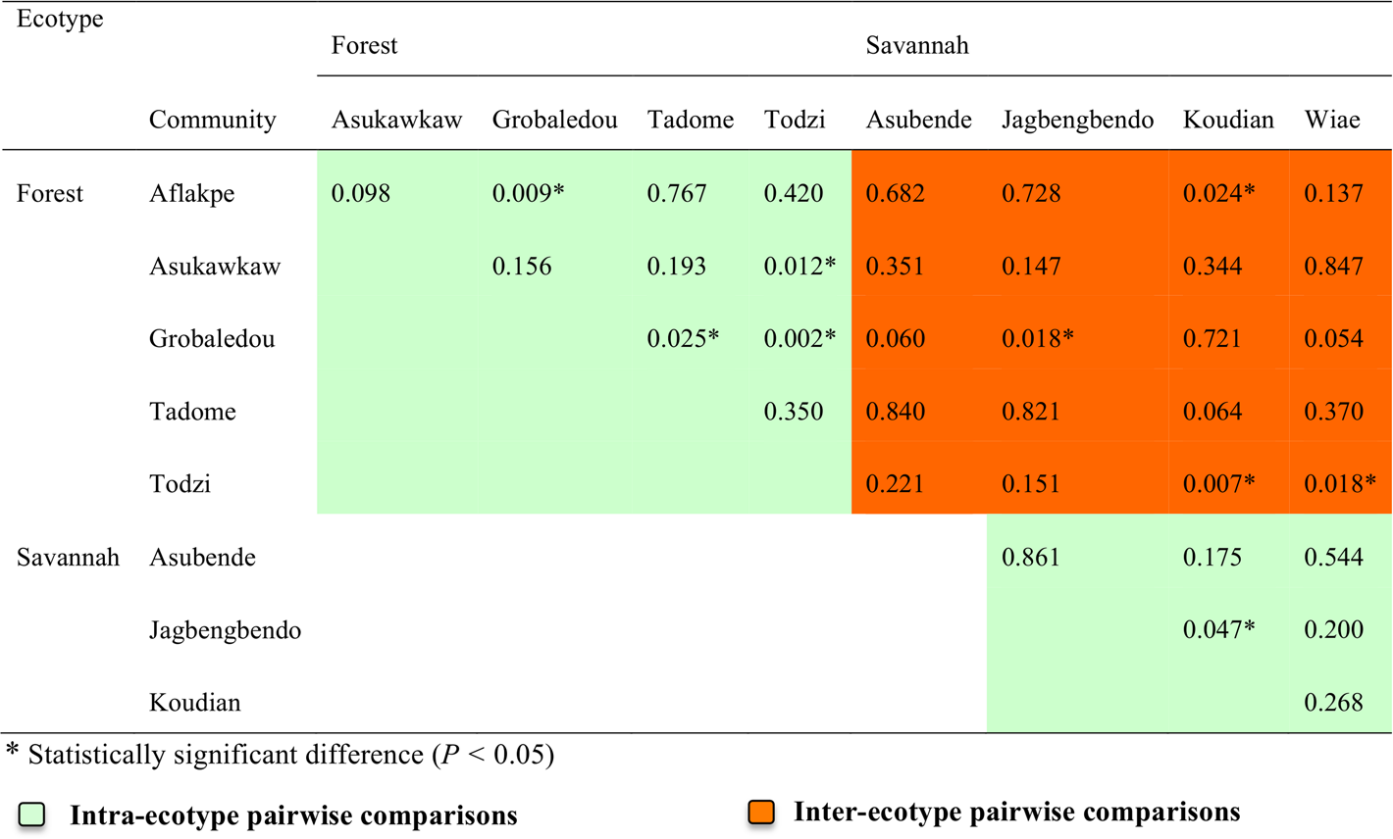
Pairwise comparison of *Wolbachia*: nuclear copy number ratios among nine study communities. *P-*values from Wilcoxon rank sum test of *Wolbachia*: nematode genomes copy number ratios of pairs of study communities are presented. *P-*values less than 0.05 were considered significant. *Green* shading represents pairwise comparisons between communities of like ecotype; *orange* shading represents pairwise comparisons between communities of differing ecotypes

The variation between communities was less extreme, although median values for most communities showed a ~ 10–100-fold range of inter-community variation. The variation among communities was, however, statistically significant (*K-W* = 18.012, *df* = 8, *P* = 0.021), and was driven mainly by 3 communities (i.e. Grobaledou, Todzi and Koudian; see Figs. 3a and 4; Additional file 4: Figure S3). The two forest communities, Todzi and Grobaledou, recorded the highest and lowest median *Wolbachia*: nuclear copy number ratios (23.46 and 1.22; approximately 20-fold variation in the median values), respectively. The strong effect of inter-community (intra-ecotype) rather than inter-ecotype variation is illustrated further by examination of the pattern of pairwise differentiation between communities: there were approximately equal numbers of intra-ecotype pairwise comparisons with *P* < 0.05 as there were inter-ecotype community comparisons with *P* < 0.05 (five and four significant pairwise comparisons for intra-ecotype and inter-ecotype, respectively; Fig. 4).

When data for forest and savannah ecotypes were aggregated, significant variation in *Wolbachia* densities within but not between ecotypes was observed (Fig. 3b). The *Wolbachia*: nematode copy number ratios for samples obtained from communities classified as forest ecotype ranged from 0.02 to 452.99, and from 0.01 to 1106.25 in the savannah ecotypes. In contrast to Higazi et al. [18], the median *Wolbachia*: nematode copy number ratio of the forest ecotype data reported here (median: 10.58) was not significantly different from that of the savannah ecotype (median: 9.10; *W* = 7,079, *df* = 1, *P =* 0.645). Higazi et al. [18] analysed worms that had been categorised as forest/non-blinding and savannah/blinding based on O-150 genotype. Other works examining the correlation between O-150 genotype, pathology and geographical origin of the parasites casts doubt on this correlation [22, 23, 33] and are more consistent with the apparent association of O-150 genotype and ecotype being a function of parasite population genetic structure rather than linked to pathogenicity *per se*. Thus, the difference in *Wolbachia*: nuclear copy number ratios observed by Higazi et al. may also be a marker of population structure rather than a marker of pathogenicity.

There was a high degree of variation of *Wolbachia*: nuclear copy number ratios within both IVM-treated and IVM-untreated populations (Fig. 3c), but there was no significant difference between the two treatment categories (*W* = 5,661, *df* = 1, *P =* 0.869). *Wolbachia*: nuclear ratios for the IVM-untreated group ranged from 0.11 to 452.99 (median: 8.81), whereas the range for the IVM-treated group was 0.01 to 1106.25 (median: 9.34). We also compared *Wolbachia*: nuclear copy number ratios with the number of rounds of IVM treatment (Additional file 5: Figure S4), and did not observe a clear relationship between ivermectin treatment duration and *Wolbachia* copy number. This contrasts with the inference from *Wolbachia* read frequency in our preliminary next-generation sequence data from pools of treated and untreated worms (Additional file 1: Table S1), which showed a difference in *Wolbachia*: nuclear genome sequencing depth between pools of treated and untreated worms. These NGS pools were sampled from different worm populations than those used for the qPCR analysis, suggesting that the different outcomes of the two analyses may be due at least in part to population structure between sampling sites.

A second NGS analysis was carried out to explore a different aspect of IVM treatment and *Wolbachia* density. In this analysis, the comparison was based on sequencing depth from whole genome NGS data of individual worms rather than sequencing depth in pools (as in the preliminary NGS data). In addition, in this analysis the comparison was between worms whose reproductive status at 90 days post-IVM administration was known and had been classified as “good responders” and “sub-optimal responders” [25–27] rather than simply IVM-treated *vs* IVM-untreated. Furthermore, these worms were sampled from Ghanaian communities that are much closer together than the sampling strategy in the previous NGS analysis (which included worms from Ghana and Cameroon), so that analysis of these worms is perhaps less likely to be confounded by population subdivision based on geographic origin. Comparison of good and sub-optimal responder worms identified a significant correlation between ivermectin response and *Wolbachia*: nuclear copy number ratios (Fig. 3d; *W* = 257, *df* = 1, *P* = 0.020), whereby sub-optimal responder worms (which have recovered some reproductive activity at day 90 post drug administration) had significantly lower *Wolbachia*: nuclear copy number ratios than worms which had responded as expected to ivermectin and remained non-reproductive at day 90. It is important to note that unlike the female *D. immitis* reported by Bazzocchi et al. [24], DNA for the *O. volvulus* sequenced here was prepared from about 4 cm of the head region of the worm which excluded any reproductive organ tissues, so the difference in *Wolbachia*: nuclear copy number ratios between good and poor responding worms is not a reflection of the presence or absence of embryos and/or microfilaria in their uteri.

Our findings suggest that differences between individual worms (intra-population) is the level at which *Wolbachia* densities vary most strongly. The existence of such extensive variation (over several orders of magnitude) between individual adult worms of both genders in all communities examined raises questions regarding the nature of the proposed symbiosis between the *Wolbachia* and their worm hosts, and the mechanisms by which *Wolbachia* density per worm is determined or regulated. If we accept the hypotheses that worms (i) require metabolic products from *Wolbachia* [11] and (ii) actively regulate *Wolbachia* density [34], how does *Wolbachia* meet the metabolic requirements of the worms in the face of such extreme variation, and why does the regulatory mechanism allow the *Wolbachia*: nuclear genome ratio to be more than 1,000 in some worms and as low as 0.01 in others? The extreme variation observed seems more consistent with stochastic variation in *Wolbachia* copy number rather than with a regulated symbiotic relationship or implies that worms are able to tolerate extreme variation in the supply of whatever *Wolbachia* metabolic products are required by adult worms. This extreme variation may, however, explain the slow and variable rate at which *Wolbachia* content declines during antibiotic treatment [35] on the assumption that *Wolbachia* clearance rate is correlated inversely with *Wolbachia* density (more *Wolbachia*, slower clearance).

The next level at which *Wolbachia*: nuclear copy number ratios clearly vary is the geographic origin of the parasite population sampled (at a community or perhaps river basin level, for example) rather than ecotype or IVM treatment history. We suggest four factors that could contribute to variation in *Wolbachia*: nuclear copy number ratios in different parasite populations reported here. First, given evidence of wide genetic variation in *O. volvulus* populations within and between endemic communities (Doyle & Grant, in preparation) and the ability of *O. volvulus* to maintain a homeostatic balance of *Wolbachia* densities [34], it is possible that there may be variation in the level at which specific worm genotypes regulate *Wolbachia* densities. Thus, given that genetic variation between worm populations exists at the community level, genetically determined differences in *Wolbachia* density would also manifest at the community level if this were the case. Second, it is possible that genetic variation between *Wolbachia* populations (rather than between worm populations) could account for the high heterogeneity of copy number. Our preliminary analysis of NGS data has provided evidence of such spatially organised *Wolbachia* genetic variation and we are in the process of exploring this variation. Third, variation in factors such genotype, diet and drug treatment history of human populations in endemic communities could contribute to the high heterogeneity of *Wolbachia*: nuclear copy number ratios observed in this study. Finally, we point out that the measurement of *Wolbachia*: nuclear copy number ratio is a single snapshot in time, and that the inter-worm variation we observed may reflect a dynamic process of temporal variation within individual worms rather than a fixed property of individuals.

## Conclusions

The data presented here are not consistent with the conclusions of a previous report [18] that parasite ecotype, and hence possibly parasite pathogenicity (blinding/savannah vs. non-blinding/forest), is correlated with *Wolbachia* density in adult worms, although we should point out that neither we nor Higazi et al. [18] report clinical data for the individual patients from whom worms were removed. We should also point out that it is not possible to compare the data reported here, which refers to individual adult worms, directly with the data reported by Higazi et al. [18], given that their measurements of *Wolbachia*: nuclear ratios were made using DNA prepared from intact nodules that contained an unknown mix of adult males, females and microfilariae. It seems reasonable to assume, however, that adults contribute the bulk of the DNA in that study. If so, the hypothesis that *Wolbachia* density per cell in adult worms is correlated with immunopathology that is discussed in that work seems unlikely also on the grounds that (a) immunopathology is provoked primarily in response to the presence of dead microfilaria in the skin and cornea [36] rather than to the presence of adult worms (which is why IVM prevents pathology despite the absence of macrofilaricidal efficacy), (b) histological data suggest that there is likely to be significantly less variation in *Wolbachia* density in microfilariae than in adults [37], and (c) the *Wolbachia* density in *B. malayi* microfilaria is lower and more constant than in adults [29] and yet there is still significant unexplained variation in pathology in lymphatic filariasis. Aside from questions relating to pathology, the findings presented here may have important practical implications for the development of antibiotics targeting *Wolbachia* as a macrofilaricidal adjunct or alternative to (microfilaricidal) IVM treatment. If, as seems reasonable, the efficacy of a given dose of antibiotic is influenced by *Wolbachia* density, then the 10^5^-fold variation in *Wolbachia* density we report here may result in variable outcomes of antibiotic treatment which may, in turn, complicate finding a single efficacious antibiotic dose for mass drug administration. Furthermore, if variation in *Wolbachia* density proves to be a determinant of variable response to antibiotic treatment and is heritable, then it is possible that selection may occur that favours survival of worms with densities of *Wolbachia* that allow escape from antibiotic treatment.

## Additional files

Additional file 1: Table S1. Number (and proportion of total) of *Wolbachia* reads from next-generation sequence (NGS) data analysis. Analysis of NGS data of IVM treated and untreated *O. volvulus* worms from Ghana and Cameroon showed higher proportions of *Wolbachia* reads in the treated worms from both Ghana and Cameroon (i.e. 6.03 and 2.46%, respectively) compared to the untreated worms from both countries (i.e. 0.90 and 0.87%, respectively). (TIF 319 kb)

Additional file 2: Figure S1. Validation of qPCR assay. **a**, **b** *wsp* plasmid and genomic DNA-derived standard curves with efficiency values of 93.5 and 92.7% respectively. **e**, **f** *gr* plasmid and genomic DNA-derived standard curves with efficiency values of 97.4 and 95%, respectively. **c**, **g** Melt peak analysis showing a single peak for both *wsp* and *gr*, inferring PCR specificity. **d** Agarose gel image showing: Lane 1: 100 bp molecular weight marker; Lane 2: positive control for *wsp* primer; Lanes 3–5: *wsp* qPCR amplicons (110 bp) from the 3 different individual *O. volvulus* worms; Lane 6: no template control. **h** Agarose gel image displaying: Lane 1: 100 bp molecular weight marker; Lane 2: positive control template for *gr* primer; Lane 3–5: *gr* qPCR products (103 bp) from 3 different individual *O. volvulus* worms; Lane 6: no template control. (TIF 1591 kb)

Additional file 3: Figure S2. *Wolbachia*: nuclear copy number ratio variation between female and male worms. Box and whisker plot shows the median (line within box), 25^th^ and 75^th^ percentile (lower and upper limits of the box respectively). The whiskers indicate the 10^th^ and 90^th^ percentiles, with outliers represented by dark dots. A Wilcoxon rank sum test was used to compare the medians between the two groups. (TIF 159 kb)

Additional file 4: Figure S3. Pairwise comparisons of *Wolbachia*: nematode genome copy number ratios among different communities. **a** Manhattan plot showing *P*-values from pairwise Wilcoxon rank sum tests between study communities. **b** Bar plot showing the number of significant associations from pairwise tests. Communities are colour-coded according to the ecotype of *O. volvulus* worms sampled. (TIF 413 kb)

Additional file 5: Figure S4. Comparisons of *Wolbachia*: nuclear copy number ratios with duration of ivermectin treatment. (TIF 214 kb)

## Abbreviations

Cq: Quantification cycle of the qPCR
*df*: Degrees of freedom
E: Amplification reaction efficiency
IVM: Ivermectin
*K-W*: Kruskal-Wallis rank sum test statistic
ng: Nanogram
NGS: Next-generation sequencing
PCR: Polymerase chain reaction
qPCR: Quantitative polymerase chain reaction
*W*: Wilcoxon rank sum test statistic
WHO: World Health Organization
μCq: Mean quantification cycle of the qPCR
