# Supplementary figures and images for: Significant heterogeneity in Wolbachia copy number within and between populations of Onchocerca volvulus
Source: Parasit Vectors. 2017 Apr 18;10:188. doi: 10.1186/s13071-017-2126-4 (PMC5395808; doi:10.1186/s13071-017-2126-4)

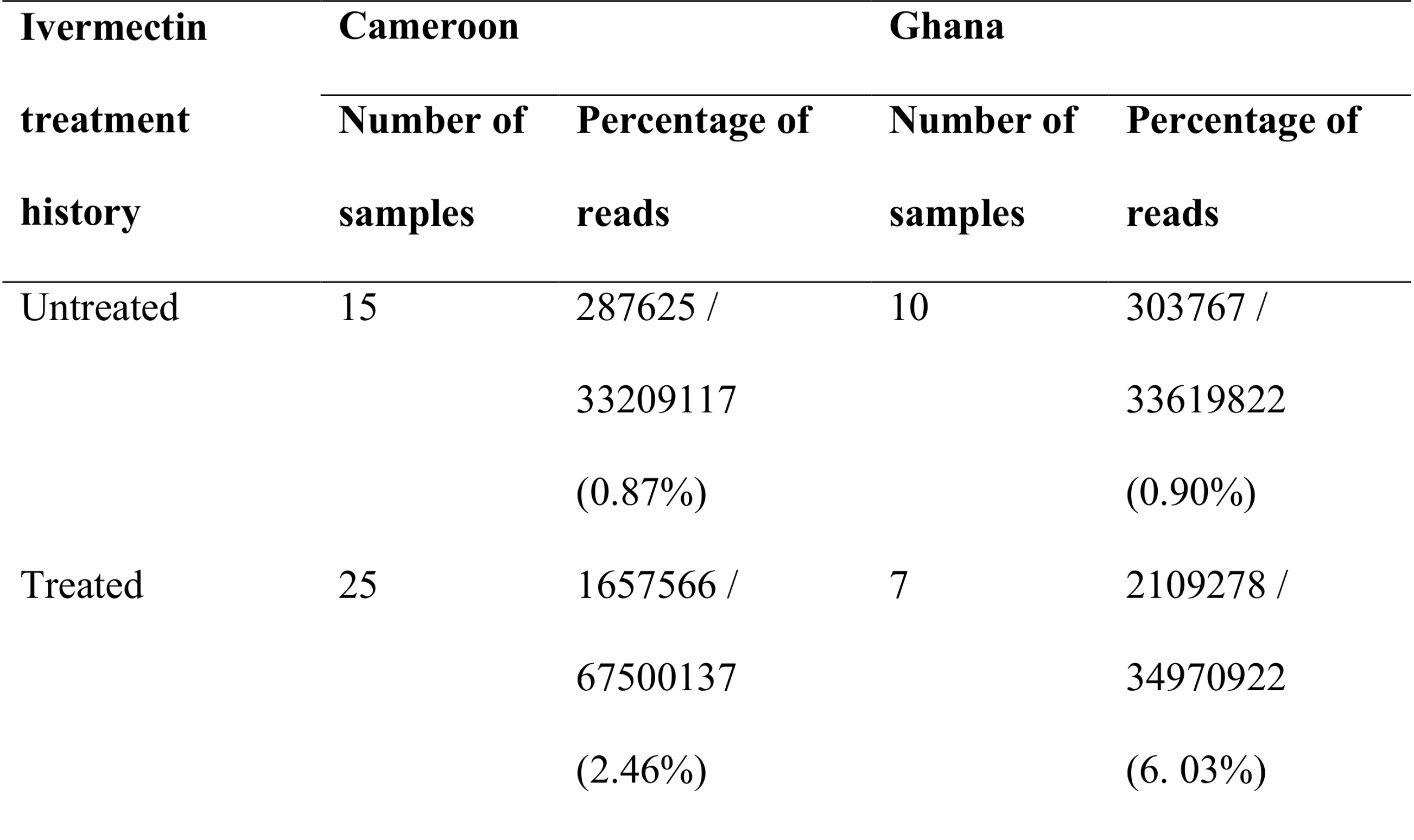

Supplement: Supplementary file 1 — Number (and proportion of total) of Wolbachia reads from next-generation sequence (NGS) data analysis. Analysis of NGS data of IVM treated and untreated O. volvulus worms from Ghana and Cameroon showed higher proportions of Wolbachia reads in the treated worms from both Ghana and Cameroon (i.e. 6.03 and 2.46%, respectively) compared to the untreated worms from both countries (i.e. 0.90 and 0.87%, respectively). (TIF 319 kb) [file 13071_2017_2126_MOESM1_ESM.tif]

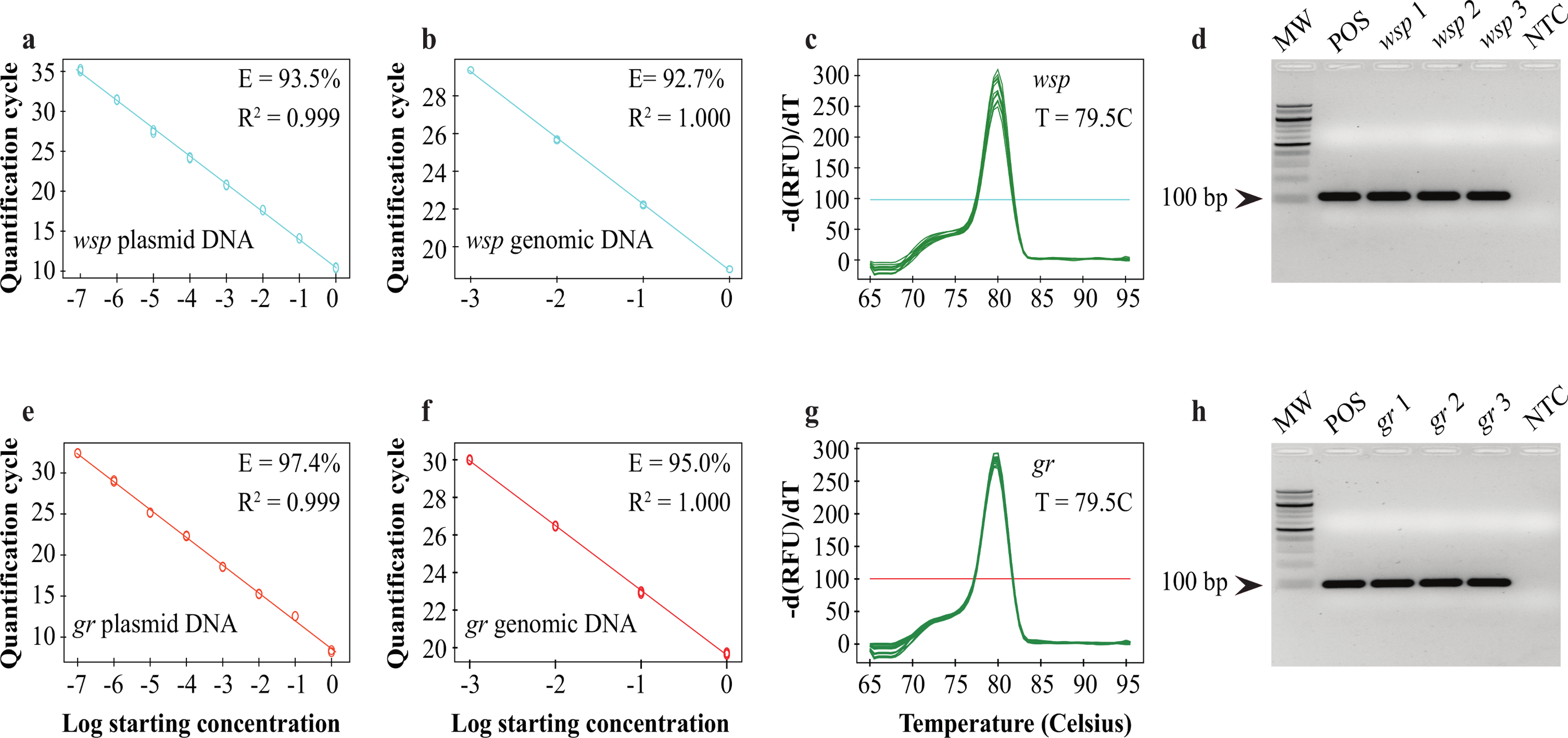

Supplement: Supplementary file 2 — Validation of qPCR assay. a, b wsp plasmid and genomic DNA-derived standard curves with efficiency values of 93.5 and 92.7% respectively. e, f gr plasmid and genomic DNA-derived standard curves with efficiency values of 97.4 and 95%, respectively. c, g Melt peak analysis showing a single peak for both wsp and gr, inferring PCR specificity. d Agarose gel image showing: Lane 1: 100 bp molecular weight marker; Lane 2: positive control for wsp primer; Lanes 3–5: wsp qPCR amplicons (110 bp) from the 3 different individual O. volvulus worms; Lane 6: no template control. h Agarose gel image displaying: Lane 1: 100 bp molecular weight marker; Lane 2: positive control template for gr primer; Lane 3–5: gr qPCR products (103 bp) from 3 different individual O. volvulus worms; Lane 6: no template control. (TIF 1591 kb) [file 13071_2017_2126_MOESM2_ESM.tif]

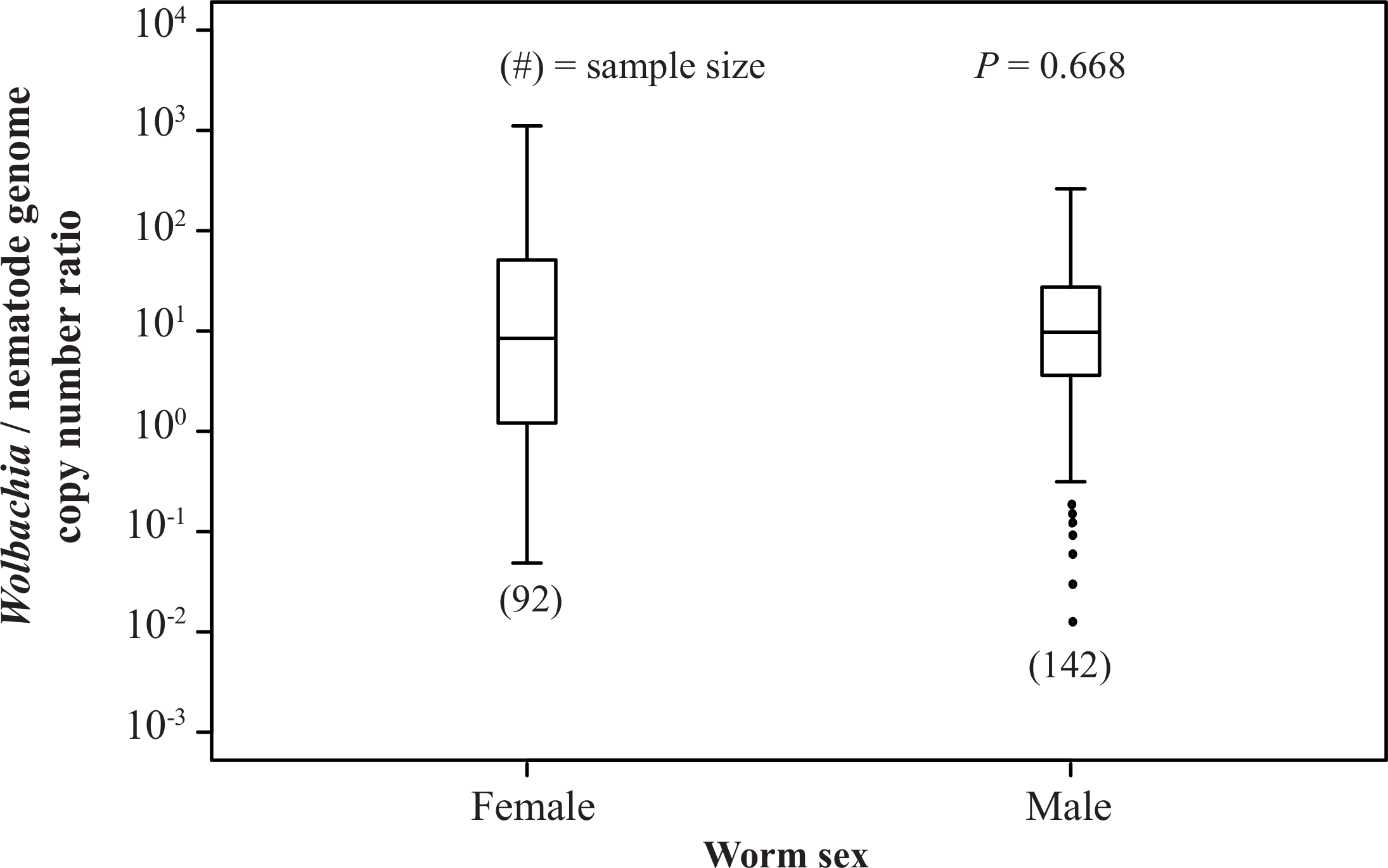

Supplement: Supplementary file 3 — Wolbachia: nuclear copy number ratio variation between female and male worms. Box and whisker plot shows the median (line within box), 25th and 75th percentile (lower and upper limits of the box respectively). The whiskers indicate the 10th and 90th percentiles, with outliers represented by dark dots. A Wilcoxon rank sum test was used to compare the medians between the two groups. (TIF 159 kb) [file 13071_2017_2126_MOESM3_ESM.tif]

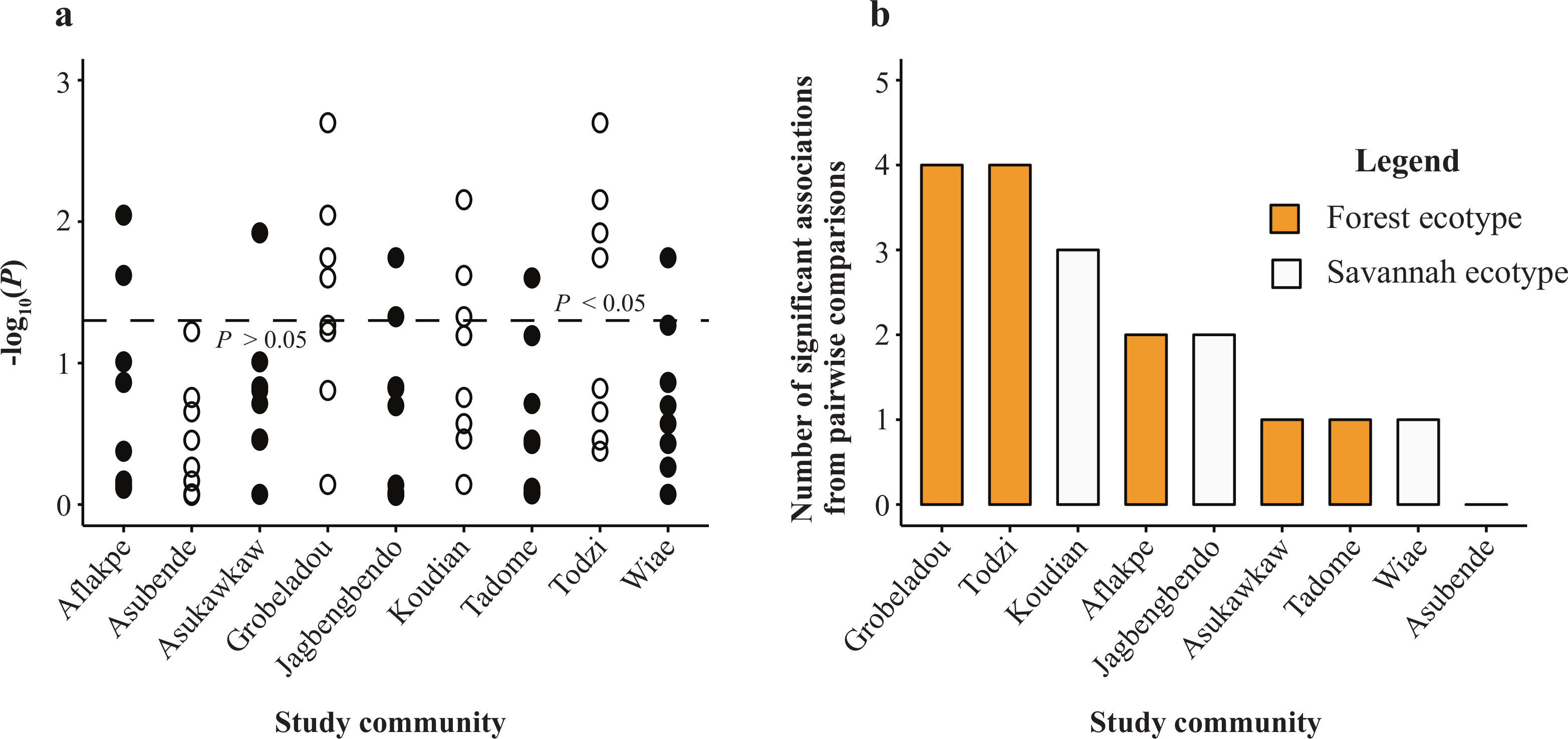

Supplement: Supplementary file 4 — Pairwise comparisons of Wolbachia: nematode genome copy number ratios among different communities. a Manhattan plot showing P-values from pairwise Wilcoxon rank sum tests between study communities. b Bar plot showing the number of significant associations from pairwise tests. Communities are colour-coded according to the ecotype of O. volvulus worms sampled. (TIF 413 kb) [file 13071_2017_2126_MOESM4_ESM.tif]

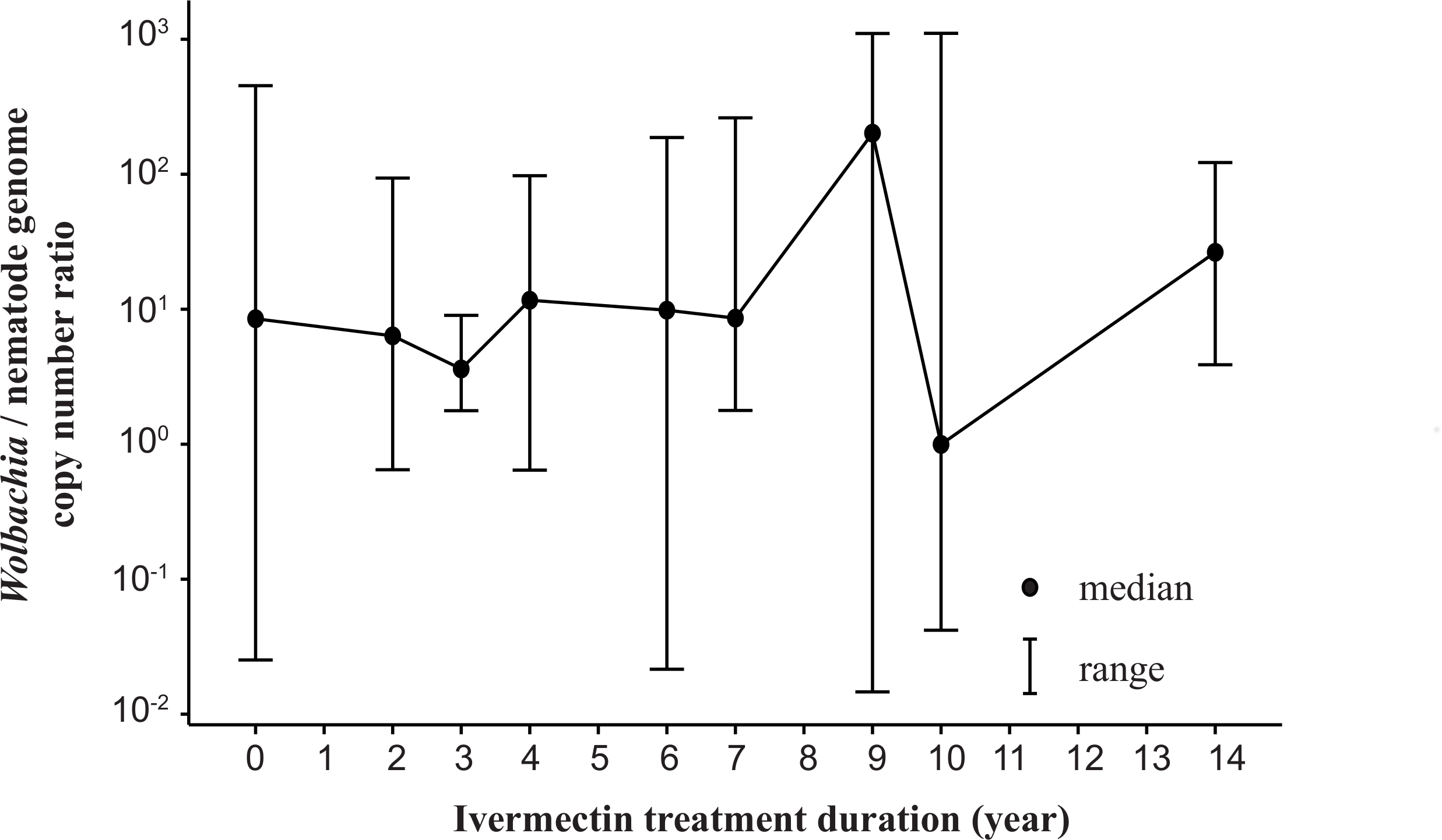

Supplement: Supplementary file 5 — Comparisons of Wolbachia: nuclear copy number ratios with duration of ivermectin treatment. (TIF 214 kb) [file 13071_2017_2126_MOESM5_ESM.tif]
